# Identifying Determinants of *Oncomelania hupensis* Habitats and Assessing the Effects of Environmental Control Strategies in the Plain Regions with the Waterway Network of China at the Microscale

**DOI:** 10.3390/ijerph110606571

**Published:** 2014-06-23

**Authors:** Juan Qiu, Rendong Li, Xingjian Xu, Chuanhua Yu, Xin Xia, Xicheng Hong, Bianrong Chang, Fengjia Yi, Yuanyuan Shi

**Affiliations:** 1 Key Laboratory of Monitoring and Estimate for Environment and Disaster of Hubei Province, Institute of Geodesy and Geophysics, Chinese Academy of Sciences, Wuhan 430077, China; E-Mails: qiujuan2011@gmail.com (J.Q.); changbianrong1220@163.com (B.C.); fengjia06@163.com (F.Y.); shiyuanyuan13@mails.ucas.ac.cn (Y.S.); 2 University of Chinese Academy of Sciences, Beijing 100049, China; 3 Hubei Provincial Center for Disease Control and Prevention, Wuhan 430079, Hubei Province, China; E-Mails: xuxj8412@foxmail.com (X.Xu); xiaxin84@163.com (X.Xia); hongxicheng@sina.com (X.H.); 4 School of Public Health & Global Health Institute, Wuhan University, Wuhan 430072, Hubei Province, China; E-Mail: yuchua@163.com

**Keywords:** *Schistosoma japonicum*, *Oncomelania hupensis*, landscape pattern analysis, environmental determinant, plain regions with waterway network, geographic information systems, microscale

## Abstract

This study aims to identify the landscape ecological determinants related to *Oncomelania hupensis* distribution, map the potential high risk of *O. hupensis* habitats at the microscale, and assess the effects of two environmental control strategies. Sampling was performed on 242 snail sites and 726 non-snail sites throughout Qianjiang City, Hubei Province, China. An integrated approach of landscape pattern analysis coupled with multiple logistic regression modeling was applied to investigate the effects of environmental factors on snail habitats. The risk probability of snail habitats positively correlated with patch fractal dimension (FD), paddy farm land proportion, and wetness index but inversely correlated with categorized normalized difference vegetation index (NDVI) and elevation. These findings indicate that FD can identify irregular features (e.g., irrigation ditches) in plain regions and that a moderate NDVI increases the microscale risk probability. Basing on the observed determinants, we predicted a map showing high-risk areas of snail habitats and simulated the effects of conduit hardening and paddy farming land rotation to dry farming land. The two approaches were confirmed effective for snail control. These findings provide an empirical basis for health professionals in local schistosomiasis control stations to identify priority areas and promising environmental control strategies for snail control and prevention.

## 1. Introduction

Schistosomiasis japonica caused by *Schistosoma japonicum* is a zoonotic disease of considerable public health and economic significance [1,2]. *Oncomelania hupensis*, the sole intermediate host of *S. japonicum*, is crucial in controlling schistosomiasis and in determining the distribution of this disease in China [3,4]. The endemic areas of *O. hupensis* in China are divided into three types: (1) plain regions with waterway networks; (2) hilly and mountainous regions; and (3) lake and marshland regions; the characteristics of these snail habitats are quite different [5]. A considerable amount of research has focused on the spatial distribution of *O. hupensis* and the prediction of its potential habitats in the latter two types of areas [6,7,8,9,10]. However, only a few studies have focused on the first type of area, which is widely distributed in the middle and lower plains of the Yangtze River basin, and knowledge on the snail distribution in this region is limited.

Over the past 50 years, the ongoing national control program (including chemotherapy, mollusciciding, health education, and sanitation and environmental improvement [11]) has made great progress in controlling schistosomiasis japonica, with a substantial reduction in the prevalence of *S. japonicum* infections in humans [12]. However, further progress in controlling this disease appeared to have stagnated [13]. Transmission has even reemerged in some areas where schistosomiasis was believed to have been eliminated [14,15]. Large-scale control strategies for extensive snail habitats are impractical; identification of areas at high risk of snail habitats and application of long-term effective measures have emerged as feasible approaches to eventually control or even interrupt schistosomiasis transmission [4,16].

Landscape epidemiology describes how the temporal dynamics of host, vector, and pathogen populations interact spatially within a permissive environment to enable transmission [17]. This epidemiology is based on the theory that most vectors, hosts, and pathogens are commonly associated with specific landscapes because environmental determinants control their distribution and abundance [18]. The concept of landscape epidemiology has been applied analytically to various vector-borne diseases, including malaria, Lyme disease, and West Nile virus [19,20]. The effects of a number of landscape metrics on the distribution of *O. hupensis* in hilly and mountainous regions have been explored [21].

We first identified the landscape ecological determinants related to the distribution of snails in plain regions with waterway networks and then mapped the potential high risk of snail habitats at the microscale. Finally, we simulated the effects of two environmental snail control strategies namely conduit hardening and paddy farming land rotation to dry farming land.

## 2. Materials and Methods

### 2.1. Study Area

**Figure 1:**
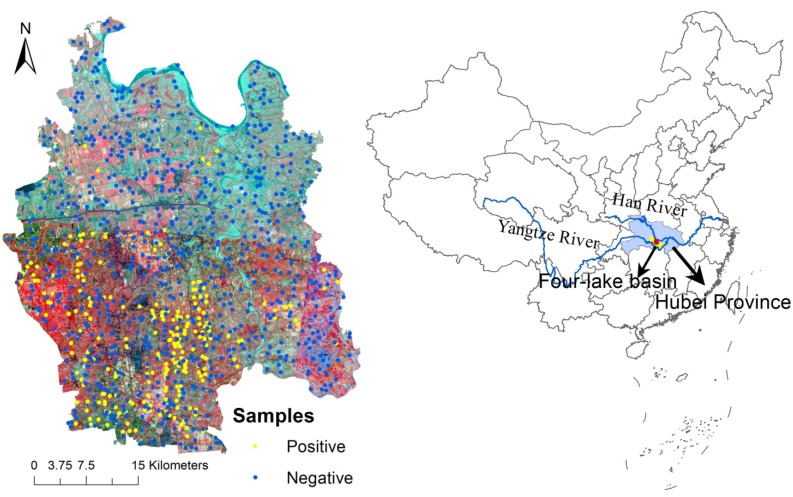
Qianjiang City location and sample distribution. Fusion and mosaic remote sensing images are overlapped.

Qianjiang City is located in the upstream of the Four-lake basin, which is a tributary of the Yangtze River middle reaches in south-central Hubei Province (Figure 1). This city is one of the most endemic areas of schistosomiasis japonica in China. In 2009, 299 schistosomiasis-endemic villages, 261 cases of advanced schistosomiasis, and 17,687 cases of chronic schistosomiasis were recorded in Qianjiang City; the prevalence rates of schistosomiasis in humans and bovines were 1.71% and 1.90%, respectively [22]. The Four-lake basin is a lacustrine-alluvial plain of the Yangtze River and Han River. The low-lying, wet, and waterlogged land, which is characterized as “land in winter, water in summer (suitable for laying eggs and hatching of snails during the dry season and for the growth of young snails during the wet season),” has created ideal snail habitats in this region. It used to be a typical lake and marshland schistosomiasis epidemic region connected with rivers and lakes, but it gradually changed into plain regions with waterway networks because of farmland reclamation from lakes and large-scale excavation channels after the mid-1970s. Accordingly, the snail distribution was linear along the ditches evolved from sheet distributions in the bottomland along the river and lakes [23,24,25,26]. In 1990, Qianjiang City had 3,662 channels, including main canals, branch canals, lateral canals, agriculture ditches, and sublateral canals, which were classified according to size and purpose; 2,122 channels had snails, accounting for 57.95% of the total number of channels and accounting for 3,092 km of the total channel length of 5,808 km [26]. Snails spread as water flowed from the main canals to other branch channels, especially when flooding occurred.

### 2.2. Data Sources

#### 2.2.1. Parasitological Data

The distribution of snails in the study area in 2010 was obtained from local schistosomiasis control stations. These data were collected by health professionals through annual snail surveys and included geo-referenced locations (latitude–longitude) of snail habitats using a global positioning system (GPS), snail reports in every village, and sketch maps of snail distribution in towns and villages. In one incidence of spatial analysis, after the incorporation of geo-referenced (latitude-longitude) snail locations, together with spatial environmental data, into a geographical information system (GIS), the snail sites were not accurately matched to their environment due to a GPS positioning error and an image registration error of the environmental data. To accurately describe the spatial distribution of snail habitat to match the environment background, a 1st order affine transformation model was employed for registrations between unregistered maps of snail distribution in towns or villages and the town or village administrative division vector layer and high-resolution remote sensing images, and then snail distribution lines were obtained through artificial digitization. Finally snail distribution line features layer were intersected with a land-use layer to generate snail distribution polygon features namely snail area. 242 snail sites (positive points) were selected in the snail area and a total of 726 non-snail sites (negative points) were randomly selected throughout non-snail area to contrast the environmental conditions associated with snail presence (positives) based on two conditions: (i) being at a minimum distance of 100 m from any positive site to prevent falling in the snail area; and (ii) being at a minimum distance of 400 m between two negative sites to avoid aggregation. The sample locations are shown in Figure 1.

Fusion and mosaic RS imaging products were used with the multispectral and panchromatic image fusion acquired from the ZiYuan 1 02c (ZY-102C) satellite (spatial resolution: 2.36 m) in 2012 as reference image when georeferencing environmental coverages and to identify little ditches (Figure 1). These products were obtained from China Center for Resources Satellite Data and Application, Beijing, China [27].

#### 2.2.2. Environmental Data

##### *Land-Use Classification* 

To obtain land-use data with high resolution and high accuracy, image segmentation based on a multiresolution segmentation algorithm and object-based classification was performed using eCognition version 8.6 software (Definiens Imaging GmbH, Munich, Germany) [28], based on the ZiYuan 102c (ZY-102C) satellite RS imaging. We compared the classified result with the 1:100,000-scale land-use data provided by the Institute of Remote Sensing and Digital Earth (RADI) under the Chinese Academy of Sciences (CAS) to ensure accuracy of classification. The main classes were water bodies (lakes, rivers, and ponds), dry and paddy agriculture land, forest land, water channel, and construction land.

##### *Landscape Pattern* 

A natural village was considered as the scale of studying the landscape pattern of *O. hupensis*. The village boundary coverage in the study area was overlaid on the land-use coverage whose features (elements) represented landscape patches and landscape metrics of each patch at the patch level. Each natural village at the landscape level was calculated using ArcGIS 10.1 (ESRI Inc., Redlands, CA, USA) and Patch Analyst version 5 [29]. Patch Analyst is an extension to the ArcView^®^ GIS system that facilitates the spatial analysis of landscape patches and modeling of attributes associated with patches. The software recognizes more than 20 landscape metrics, which can be stratified into six types: area metrics, patch density and size metrics, edge metrics, shape metrics, diversity metrics and core area metrics. A mathematical definition of each metric is detailed in reference [30]. Multicollinearity among these metrics does not increase the new information when multiple metrics are involved simultaneously, especially those that belong to the same type [31]. Basing on their ecological significance and avoiding information redundancy, we selected the following landscape metrics: fractal dimension (FD), median patch size (MedPS), patch size coefficient of variance (PSCOV), mean patch fractal dimension (MPFD), and area-weighted mean shape index (AWMSI).

##### *Normalized difference vegetation index (NDVI) and wetness index (WI)* 

NDVI and WI were extracted from Landsat-5 TM image using ERDAS IMAGINE 2013 software (ERDAS Inc., Atlanta, GA, USA). The image covering the study area with a spatial resolution of 30 m was acquired in July 2010 and downloaded from the U.S. Geological Survey (USGS [32]). The snail habitats were mainly distributed in the areas with NDVI value on some intervals [7,33,34]. To consider the non-linearity effect of NDVI, the continuous scale risk factor variable was categorized into two levels that represent the terciles of the data distribution. NDVI < 0.29 (5% tercile) or NDVI > 0.57 (85% tercile) was recoded under category 1 and NDVI ≥ 0.29 and NDVI ≤ 0.57 were recoded under category 0.

##### *Soil texture* 

The proportion of silt in the soil was selected to represent the soil texture acquired from RADI under CAS.

##### *Topological data* 

The elevation data (digital elevation model; DEM) were based on the USGS GTOPO30 Digital Dataset provided by the International Scientific and Technical Data Mirror Site, Computer Network Information Center, CAS [35].

All environmental variables were registered with reference image and carried out at a spatial resolution of 5 m.

### 2.3. Statistical Analysis

Multiple logistic regression was used to identify significant environmental covariates associated with snail distribution. Univariate logistic regression analyses were initially conducted to examine the effect of each variable on snail distribution. The relationship was evaluated by the mean odds ratios (ORs), 95% confidence intervals (CIs) of ORs, and *p* value. Variables with *p* values <0.1 were included for further analyses. Variables with relatively high collinearity were excluded, as assessed by examining the correlation coefficient and the variance inflation factors (VIFs). VIFs < 10 were considered to have low co-linearity [36]. The remaining significant independent variables were carried out in multiple backward-logistic regression to construct a final model with a significance level of *p* < 0.05. The goodness-of-fit model was evaluated by the Hosmer–Lemeshow test [37]. The area under the curve (AUC) of the receiver operating characteristic (ROC) plots was used to indicate model performance. AUC, a quantitative measure of the overall fit of the model, varies from 0.5 (chance event) to 1.0 (perfect fit) [38]. To predict the risks of snail habitats, a grid map was created using ArcGIS 10.1 on the basis of the predictive model derived from the multivariate logistic regression analysis.

Basing on the significant risk factors determined from the logistic regression models, we reduced the proportion of paddy agriculture land in villages by 80% and lowered the NDVI and WI values equal to those of concrete surface to simulate the effects of two environmental control strategies, namely, paddy farming land rotation to dry farming land and conduit hardening, in Haokou, Qianjiang City.

## 3. Results

The significant risk factors identified by logistic regression models were FD, MedPS, PSCOV, MPFD, AWMSI, dry farm land proportion, paddy farm land proportion, silt proportion, DEM, WI, and categorized NDVI (Table 1). Five factors were maintained in the multivariate analysis (*p* < 0.05). Positive effects were exhibited by FD [OR 1.359 (95% CI: 1.292–1.430)], paddy farm land proportion [OR 1.055 (95% CI: 1.032–1.079)], and WI [OR 1.052 (95% CI: 1.017–1.089)]. Significant negative effects were exerted by categorized NDVI [OR 0.351 (95% CI: 0.166–0.743)] and DEM [OR 0.886 (95% CI: 0.828–0.947)]. This result indicated that reduced categorized NDVI and elevation combined with increased FD, paddy farm land proportion, and WI increased the risk probability of snail habitats. The Hosmer and Lemeshow goodness-of-fit chi-squared test of the model was 6.46 (*p* = 0.60). The ROC curve assessed the predictive power of the model, and the AUC was 0.986 (95% CI: 0.981–0.992). The final prediction model for the risk of snail habitats was as follows:

Predictive risk of snail habitats = 1/{1 + Exp[−(−45.514 − 0.121 × DEM + 0.051 × Wetness − 1.047 × NDVI + 0.054 × Proportion paddy farm land + 0.307 × FD)]}.

**Figure 2:**
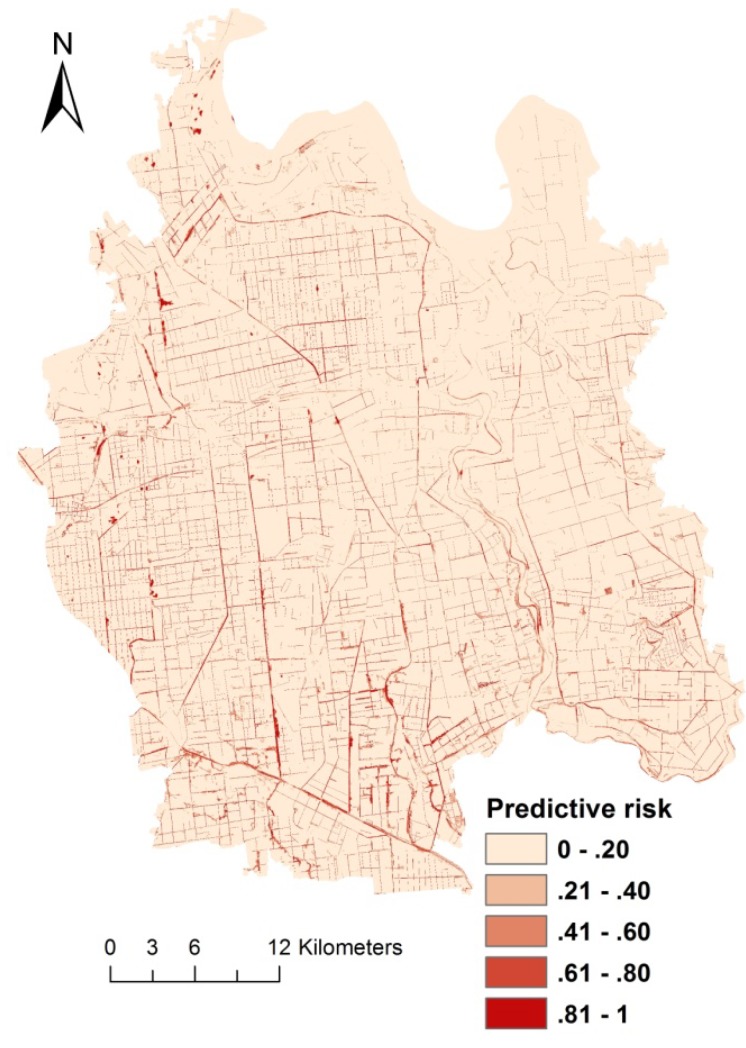
Predictive risk map of snail habitats in Qianjiang City.

**Figure 3:**
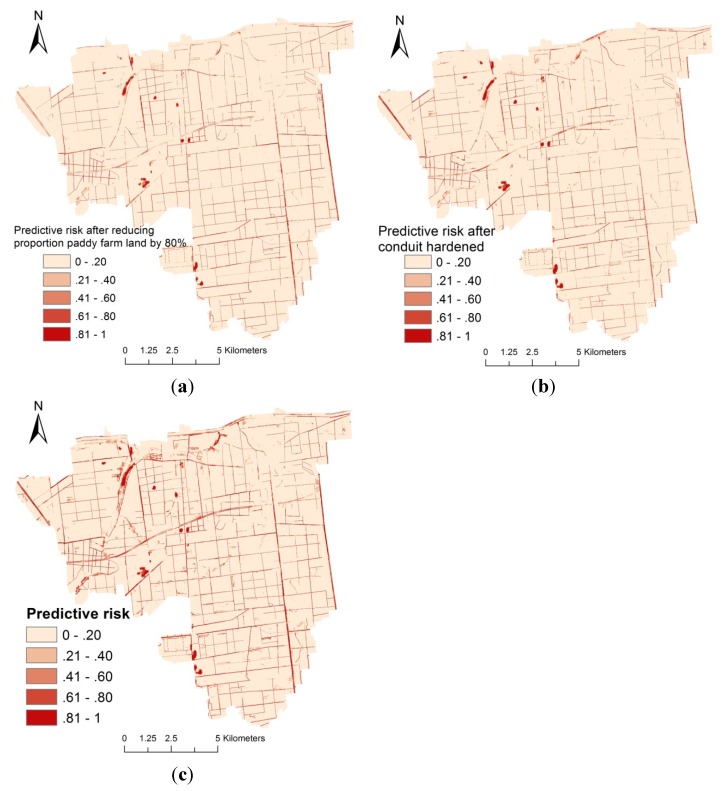
Map-simulated effects of paddy farming land rotationto dry farming land (**a**) and conduit hardening (**b**) compared with the risk before the implementation of these two environmental control strategies (**c**) in Haokou, Qianjiang City.

A predictive risk map of snail habitats was established for the study area on the basis of the final multiple logistic regression analysis (Figure 2). High-risk areas were mainly located in river beaches and crisscrossed ditches. The risk map after paddy farming land rotation to dry farming land (Figure 3a) and conduit hardening (Figure 3b) was compared with that before these two environmental control strategies were implemented (Figure 3c). The predicted risk had a significant reduction in area where the proportion of paddy agriculture land in villages reduced by 80% and the NDVI and WI values lowered equal to those of concrete surface. To quantify the effects, we collected 200 samples in the area with altered environmental factors (paddy agriculture land proportion, NDVI, and WI) and with original predictive risk (Figure 3c) higher than 0.5. 

**Table 1:** Results of the logistic regression models applied to snail habitats.

Factors	Univariate Analysis			Multivariate Analysis		
	*B*	OR (95% CI)	*p* value	*B*	OR (95% CI)	*p* value
FD (×100)	0.280	1.323 (1.272–1.377)	<0.001	0.307	1.359 (1.292–1.430)	<0.001
MedPS	0.974	2.648 (1.803–3.891)	<0.001			
PSCOV	−0.002	0.998 (0.996–1.000)	0.040			
MPFD	−11.872	0.000 (0.000–0.008)	0.001			
AWMSI	−0.450	0.637 (0.425–0.956)	0.029			
Dry farm land proportion (%)	−0.010	0.990 (0.982–0.999)	0.035			
Paddy farm landproportion (%)	0.040	1.041 (1.031–1.051)	<0.001	0.054	1.055 (1.032–1.079)	<0.001
Silt proportion (%)	0.213	1.237 (1.170–1.309)	<0.001			
DEM (m)	−0.052	0.949 (0.928–0.971)	<0.001	−0.121	0.886 (0.828–0.947)	<0.001
Wetness	0.017	1.017 (1.003–1.031)	0.017	0.051	1.052 (1.017–1.089)	0.003
NDVI	−1.358	0.257 (0.182–0.364)	<0.001	−1.047	0.351 (0.166–0.743)	0.006

The predictive risk decreased by average values of 0.22 and 0.35 and the predictive risk of 32.5% and 48.5% of the samples dropped to less than 0.5 after the proportion of paddy agriculture land in the villages was reduced by 80% and after NDVI and WI were lowered to levels equal to those of concrete surfaces, respectively.

## 4. Discussion

Numerous studies used GIS and RS combined with statistical methodologies to predict schistosomiasis infection or intermediate host snails of schistosomiasis distribution risks [4,7,9,21,39,40,41,42]. These studies served as decision-making references for schistosomiasis prevention and control strategies, such as macroscopic resource allocation. However, these studies often have limited functions as practical guides on the effective implementation of interventions for the health professionals of local schistosomiasis control stations. Their limited functions can be attributed to the low spatial resolution of selected environmental data and parasitological data based on the summarized schistosomiasis data in the administrative region (village, town, county, city, or province). The present study used high-spatial-resolution environmental data and landscape pattern analysis to identify the environmental determinants affecting the geographical variation of snail distribution. The predicted map of the snail habitat risk presented more detailed information than survey data and sketch maps. It also provided an empirical basis for identifying priority areas where implementing snail control and preventive treatment in local regions.

All observed environmental determinants related to snail distribution were interpretable and consistent with the biology of snails. Snails inhabit areas are with low relative elevation [5]. Qiangjiang City has a high terrain in the northeast and a low terrain in the southwest, and this situation pertains to the higher distribution of snails and thus higher *Schistosoma* epidemics in the southwest than in the northeast [43]. Wetness (or humidity) and vegetation are important environmental factors for *O. hupensis* prediction at the microscale [3,44]. Our study found that on the microscale higher wetness is favourable to snail survival. The categorized NDVI was shown to have a negatively effect on snail risk probability, suggesting that a moderate NDVI can increase the risk probability. This finding contradicts the results of previous studies [9,21,45]. This inconsistency in results can be attributed to the micro-level spatial scale of the present study. At the macroscale, such as town, province, or country, a higher NDVI indicating relatively higher vegetation cover possibly increases the probability of potential snail habitats compared with non-vegetation cover. At the mesoscale, such as village, a low NDVI combined with high wetness values indicates the presence of water and thus a high probability of suitable snail habitats [21]. However, at the microscale, high NDVI usually indicates the presence forest land, agricultural land, or emergent aquatic plant during the vegetative period, whereas low NDVI usually indicates the presence of water bodies or building land. Only moderate NDVI indicates appropriate abundance of green vegetation, especially weeds, which are suitable for snail habitats. This phenomenon reveals that the function of the same environmental variable varies per study scale.

The agricultural structure is important in relation to snail distribution and control [46]. The results of multiple logistic regression analysis indicated that snail risk increased with increasing proportion of paddy fields. Therefore, paddy–upland rotation or paddy farming land rotation to dry farming land can significantly reduce the probability of snail habitats and infected patients. The landscape pattern analysis and logistic regression models exhibited that decreasing FD can decrease the probability of snail habitats. High FD arises when the patch shape considerably deviates from the Euclidean geometry [47]. In plain regions, high FD can indicate irregular features, such as irrigation ditches. The positive significant association between FD and snail habitat probability can be understood because snails tend to cluster along irregular features, particularly irrigation ditches, and are less attracted to relatively flat and regular regions, such as crop fields.

Environmental modification is an effective approach for snail control to markedly reduce the prevalence of schistosomiasis in China [48,49,50,51]. Our simulated study confirmed that conduit hardening and paddy farming land rotation to dry farming land are potentially effective measures in plain regions with waterway networks because they radically change the snail habitat conditions by destroying the ecological environment of snails. These measures also reduce the probability of humans and farm cattle coming into contact with the contaminated water, consequently decreasing the chance of humans and cattle being infected with schistosomiasis [52]. The two methods had been previously implemented in some areas and obtained achievements, the snail distribution area dropped significantly [49,53,54]. However, for conduit hardening, snail reemergence indicates that maintaining and consolidating the achievements obtained necessitate further efforts to prevent hardened ditch dilapidation (e.g., collapse, crevice, and regrowth of moss and weed) and require regular desilting, weeding, and repairing of cracks and collapse to strengthen the maintenance of hardened ditches [15,55]. Moreover, environmental modification is not widely used to control snails mainly because of its higher cost and worse cost-effectiveness than mollusciciding [56]. However, mollusciciding not only requires repeated spray applications, but also pollutes the environment. Environmental modification saves resources because of its long-term stable effect and also produces no environmental pollution. Therefore, this approach is worth further implementation.

The limitations of this study are as follows: first, our prediction model ignored the spatial autocorrelation, but it’s appropriate when mapping disease over small areas, but questionable over wide areas, such as a country [57]. For snail distribution, spatial autocorrelation is anisotropic because of snail dispersal following water along ditches. Investigating spatial autocorrelation in different geographic directions is challenging and needs to be considered in further studies. Second, the weight of patch FD would be overestimated so that linear features, such as roads, might be treated as high risk area, and more representational explanatory variables should also be considered in further studies. Third, random sampling error of non-snail sites could affect model precision, but the large sample (726 non-snail sites) would make up this shortfall. A fourth limitation is the limited temporal scope of the data that doesn’t account for possible inter-annual changes to snail abundance and distribution.

## 5. Conclusions

This study investigated snail habitats in plain regions with waterway networks where endemic schistosomiasis remains an important public health problem. It provided an efficient approach to identifying environmental determinants using landscape pattern analysis combined with multiple logistic modeling at the microscale. The simulated effects of two environmental control strategies based on observed significant risk factors revealed that environmental modification is a potentially useful approach for snail control. Our predictive risk map could serve as a practical guide for the effective implementation of interventions for health professionals in local schistosomiasis control stations.
